# Association Between Eyelid Twitching and Digital Screen Time, Uncorrected Refractive Error, Intraocular Pressure, and Blood Electrolyte Imbalances

**DOI:** 10.7759/cureus.69249

**Published:** 2024-09-12

**Authors:** Irfan B Gunes

**Affiliations:** 1 Ophthalmology, Kocaeli Health and Technology University, Medical Park Kocaeli Hospital, Kocaeli, TUR

**Keywords:** intraocular pressure, electrolyte imbalance, myokymia, refractive error, eyelid twitching, digital screen

## Abstract

Introduction: Previous studies have shown that isolated eyelid myokymia (EM) is usually caused by stress, fatigue, and caffeine consumption. The purpose of this study was to evaluate the association between EM and digital screen time, uncorrected refractive error, intraocular pressure (IOP), and blood electrolyte levels.

Methods: Between February 2023 and June 2024, 103 eyes of 103 patients who applied to the ophthalmology outpatient clinic with complaints of eyelid twitching lasting for more than two weeks and 103 eyes of 103 healthy individuals as a control group were included in the study. All participants were asked to record their daily time spent with digital screens for two weeks. Cycloplegic refractive error, IOP, optic nerve head cup/disc (C/D) ratio, and blood calcium, sodium, potassium, and magnesium levels were recorded and compared between the two groups.

Results: Mean digital screen time was 4.84±1.74 hours in the control group and 6.88±2.01 hours in the EM group. It was found that digital screen time was significantly higher in the EM group compared to the control group (p<0.001). There was a strong positive correlation between the duration of eyelid twitching and the time spent in front of digital screens (p<0.001, r=0.670). There was no significant difference in cycloplegic refractive error, IOP, C/D ratio, and blood electrolyte levels between the two groups (p>0.05).

Conclusion: Prolonged digital screen time might play a role in the development of EM. On the other hand, no relationship was found between eyelid twitching and uncorrected refractive error, glaucoma, or blood electrolyte levels.

## Introduction

On a daily basis, numerous individuals present themselves at the ophthalmology and neurology outpatient clinics with complaints of involuntary contractions of the eyelids. As a consequence of their research, many patients are concerned that glaucoma, neurological diseases, and muscle diseases may be the cause of this condition. Eyelid myokymia (EM) is defined as irregular contractions of the orbicularis oculi muscle, often unilateral and localized in the lower eyelid [1]. The precise mechanism and cause of its occurrence are not fully understood. However, stress disorders, chronic fatigue, irregular eating habits, and excessive caffeine consumption have been identified as potential contributing factors [1,2]. EM is commonly referred to as eye twitching and is a benign condition that typically resolves spontaneously within days, particularly with rest [3,4]. In some patients, this condition persists for months, significantly reducing the quality of life of the patient. Botulinum toxin applied to the orbicularis oculi muscle is an effective treatment for prolonged eyelid twitching [3,5,6]. In addition to botulinum toxin, various other substances have been trialed as systemic treatments, including calcium (Ca), phosphorus, magnesium (Mg), tonic water, and vitamins [7].

Focusing on a digital screen for an extended period of time can cause the eyelids to squint, particularly due to the effect of screen light. Furthermore, individuals with an uncorrected refractive error create a pinhole effect by squinting their eyelids in an attempt to enhance visual acuity. It is hypothesized that prolonged squinting of the eyelids during the day may result in fatigue and involuntary contractions of the orbicularis oculi muscle. To the best of my knowledge, no studies in the literature have investigated the relationship between digital screen time, uncorrected refractive error, and long-standing eyelid twitching.

In this study, digital screen time, uncorrected refractive error, intraocular pressure (IOP), and blood electrolyte levels of patients with long-standing eyelid twitching were compared with those of a healthy control group.

## Materials and methods

This cross-sectional case-control study was conducted in accordance with the principles of the Declaration of Helsinki and was carried out with the approval obtained from the Local Ethics Committee for Clinical Practices at Kocaeli Health and Technology University (number: 72-21/05). Prior to their inclusion in the study, all participants were required to provide written informed consent.

A total of 128 patients who presented to the ophthalmology outpatient clinic with complaints of twitching of the eyelids were included in the study between February 2023 and June 2024. Additionally, 103 healthy participants of a similar age group and gender distribution with no eye complaints were included in the control group. Best-corrected visual acuity, anterior segment examination findings obtained via slit lamp microscopy, and posterior segment examination findings obtained with a 90D lens were recorded. The IOP was quantified with Goldmann applanation tonometry.

In order to determine mean daily digital screen time, total work hours, daily sleep time, and coffee consumption, patients presenting with eyelid twitching for a duration exceeding two weeks were provided with a chart to be completed upon their initial visit to the outpatient clinic (see Table 3 given in Appendices). Furthermore, healthy participants were provided with a chart to complete upon enrollment in the study. At the conclusion of the two-week period, the completed charts were collected from the individuals. In order to exclude any potential effects of chronic fatigue and excessive coffee consumption, the study included 103 patients and 103 healthy participants, all of whom had an average daily working time of less than nine hours, an average daily sleep time of more than seven hours, and an average daily coffee consumption of less than two cups. The participants were requested to record the amount of time they spent utilizing their cell phones, tablets, and computers throughout the day in hours, as a measure of their digital screen time.

All patients were referred to the neurology clinic for consultation, and all participants were investigated in the national health registry system for previous diagnoses of systemic diseases.

The study included patients older than 18 years of age with eyelid twitching for longer than two weeks and participants who gave consent for the determination of blood electrolyte levels. Individuals under the age of 18, those who wore glasses or contact lenses due to refractive error, patients with amblyopia or dry eye disease, long-term eye drop users, glaucoma patients, patients with known systemic diseases and systemic drug users, and those who consume energy drinks were excluded from the study. Eight patients (6.2%) were excluded due to excessive coffee consumption, seven patients (5.4%) due to long working hours, and 10 (7.8%) due to insufficient sleep time.

**Figure 1:**
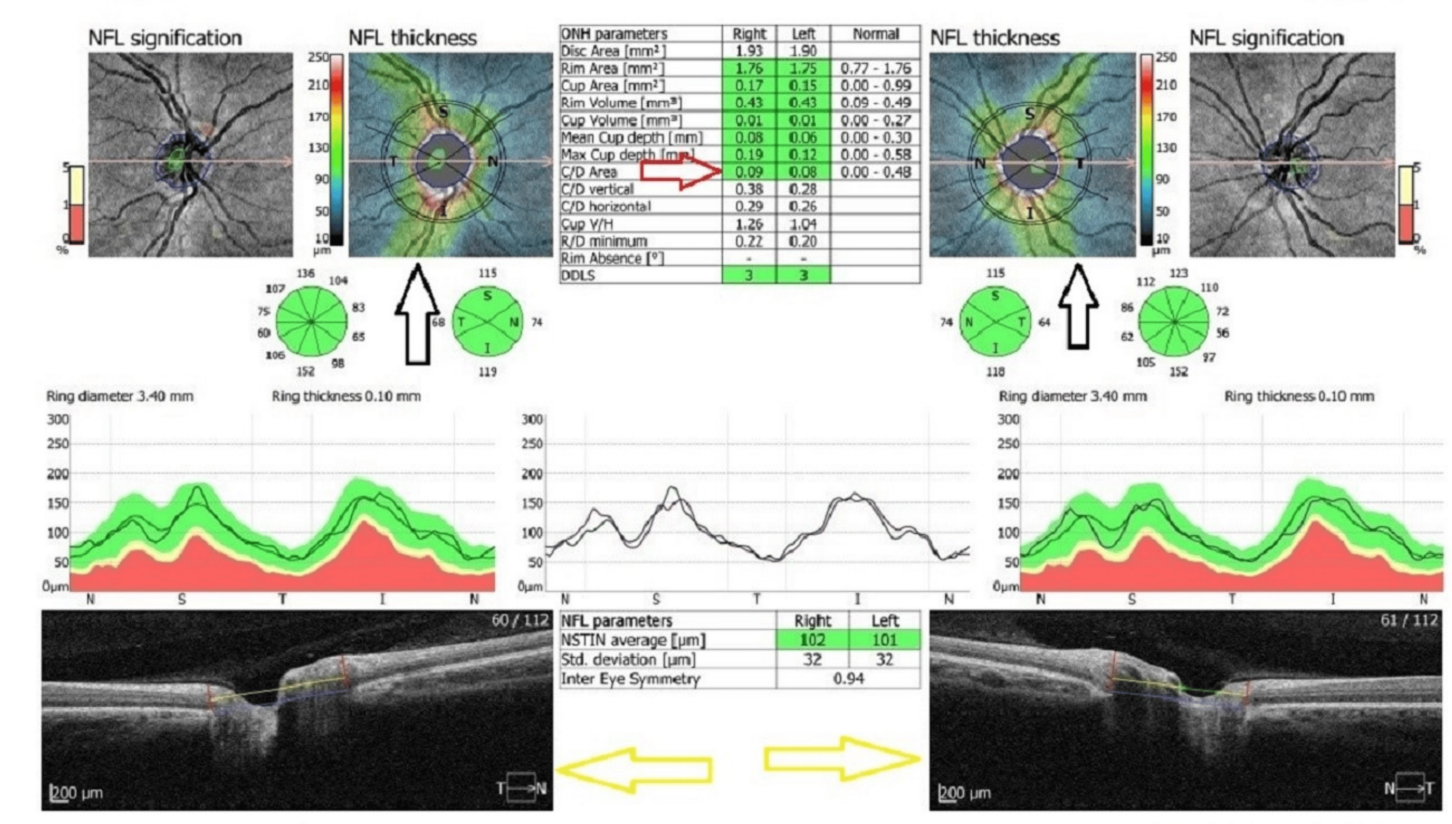
Measurement of the C/D area ratio of the optic nerve head with OCT. The OCT scan, with a quality score of 9/10, shows cross-sectional images of the optic disc for the right and left eyes. The yellow arrows show the cross-sectional image of the optic disc of the right and left eye. Black arrows show the automatically drawn image of the C/D boundaries of the optic nerve head. The red arrow indicates the numerical value of the C/D area ratio of the optic nerve head for the right and left eye. OCT: Optical coherence tomography, NFL: Nerve fiber layer, C/D: cup/disc

To evaluate the participants for the presence of glaucoma, the cup/disc (C/D) ratio of the optic nerve head was automatically measured and recorded in disc acquisition mode of an optical coherence tomography (OCT) device (Optopol Technology, Zawiercie, Poland) (Figure 1).

Prior to the measurement of refractive error, cyclopentolate 1% (Sikloplejin, Abdi Ibrahim, Istanbul, Turkey) was instilled in both eyes of the participants twice, with an interval of five minutes. Cycloplegia was deemed to have occurred in patients with a pupil diameter exceeding 6 mm and the absence of a light reflex 30 minutes following the second drop. The cycloplegic refractive error was measured using an autorefractometer (Full Auto Ref-Keratometer RK-F2; Canon, Kanagawa, Japan). The mean of three consecutive measurements was accepted as the cycloplegic refractive error value. The spherical equivalent (SE) value of the participants was calculated using the formula: SE = spherical refractive error + 0.5 x cylindrical refractive error. The blood calcium (Ca), sodium (Na), potassium (K), and magnesium (Mg) values of all participants were recorded.

The mean SE, mean digital screen time, C/D ratio, and blood electrolyte values of the patient group were compared with those of the control group.

Statistical evaluation of the data was performed using IBM SPSS for Windows version 20.0 (IBM Corp., Armonk, USA). Descriptive statistics for categorical variables were presented as numbers and percentages, and numerical variables were presented as mean ± standard deviation and minimum-maximum values. In the analysis of numerical data, conformity to normal distribution was examined with the Kolmogorov-Simirnov test. The median difference between the two independent groups was analyzed with the independent samples t-test. Spearman coefficient was used in correlation tests. Data were analyzed at 95% confidence level and tests were considered significant if p-value was less than 0.05.

## Results

The study included 103 eyes of 103 patients aged 19-62 years with eyelid twitching for more than two weeks as the EM group and 103 randomly selected eyes of 103 healthy participants aged 19-60 years as the control group. In the EM group, the eye with the most pronounced twitching was included in the study. There was no significant difference between the groups with regard to age and gender distribution (p=0.102 and p=0.675, respectively) (Table 1). In the EM group, the mean IOP was 15.96±3.05 mmHg, while the mean C/D was 0.25±0.11. In the control group, the mean IOP was 15.62±3.00 mmHg and the mean C/D was 0.23±0.12. There was no statistically significant difference between the mean IOP and C/D values between the groups (p=0.422 and p=0.362, respectively). The mean daily digital screen time was 6.88±2.01 hours in the EM group and 4.84±1.74 hours in the control group. The digital screen time was found to be significantly higher in the EM group compared to the control group (p<0.001). The mean spherical equivalent value, calculated to evaluate the uncorrected refractive error of the participants, was 0.33±0.42 in the EM group and 0.31±0.40 in the control group. The mean SE value was not significantly different between the EM group and the control group (p=0.784) (Table 1).

**Table 1:** Demographic data, ocular findings, and digital screen timing of the groups. Descriptive characteristics were given as mean ± standard deviation (SD) (minimum-maximum). A p-value <0.05 was accepted as statistically significant. n: Number of cases, M/F: Males/females, IOP: Intraocular pressure, C/D: cup/disc, SE: Spherical equivalent, ^a^: Independent t-test, ^b^: Chi-square test

Variable	Eyelid Myokymia Group n:103	Control Group n:103	p
Age (years)	33.33±7.93 (19-62)	35.18±8.26 (19-60)	0.102^a^
Gender (F/M)	57/46	54/49	0.675^b^
Complaint duration (weeks)	3.73±1.33 (2-12)	-	-
IOP (mmHg)	15.96±3.05 (12-22)	15.62±3.00 (11-22)	0.422^a^
C/D ratio	0.25±0.11 (0.09-0.41)	0.23±0.12 (0.11-0.39)	0.362^a^
Digital screen timing (hours)	6.88±2.01 (1-11)	4.84±1.74 (1-9)	<0.001^a^
SE value	0.33±0.42 (0-2.50)	0.31±0.40 (0-2.25)	0.784^a^

Table 2 presents the mean daily sleep time, working time, coffee consumption, and blood Na, Ca, K, and Mg levels of the EM group and control group. No significant differences were observed in the blood electrolyte levels between the two groups (p>0.05). Two of the participants with eyelid twitching had hypernatremia and three had hypocalcemia. In the control group, two individuals had hypocalcemia. The mean daily sleep duration, working hours, and coffee consumption of participants in the EM group and control group were found to be similar (p>0.05) (Table 2).

**Table 2:** Mean daily sleep time, working time, coffee consumption, and blood electrolyte levels of the eyelid myokymia group and the control group. Descriptive characteristics were given as mean ± standard deviation (SD) (minimum-maximum). A p-value <0.05 was accepted as statistically significant. n: Number of cases, Na: Sodium, Ca: Calcium, K: Potassium, Mg: Magnesium, ^a^: Independent t-test

Variable	Eyelid Myokymia Group n:103	Control Group n:103	p
Sleeping time (hours/day)	7.88±1.38 (6-11)	8.02±1.30 (6-11)	0.437^a^
Working time (hours/day)	8.18±0.57 (7-11)	8.14±0.63 (7-11)	0.645^a^
Coffee consumption (cups/day)	0.97±0.59 (0-2)	0.88±0.73 (0-2)	0.318^a^
Blood Na levels (mmol/L) Ref: 135-145	139.61±3.16 (131-152)	140.26±3.51 (133-150)	0.164^a^
Blood Ca levels (mg/dL) Ref: 8.4-10.2	9.19±0.50 (8.6-10.1)	9.28±0.49 (8.5-9.9)	0.189^a^
Blood K levels (mmol/L) Ref: 3.5-5.5	4.64±0.37 (3.8-5.1)	4.61±0.36 (3.7-5.0)	0.534^a^
Blood Mg levels (mg/dL) Ref: 1.6-2.6	1.95±0.28 (1.5-2.9)	1.97±0.27 (1.7-2.6)	0.507^a^

A significant strong positive correlation was observed between the duration of digital screen time and the duration of complaints in the EM group (p<0.001, r=0.670).

## Discussion

EM, also known as eyelid twitching, represents one of the most frequent reasons for patients to present at outpatient clinics of ophthalmology and neurology. A paucity of studies exists on the prevalence of EM. In studies on its etiology, the frequently emphasized causes are stress, anxiety, fatigue, and caffeine consumption [1,8]. Patients who frequently describe unilateral and localized involuntary contractions in the lower eyelid are concerned that these contractions may be caused by glaucoma, refractive error, low blood electrolytes, especially Mg level, or neurological disease. Although there are studies in the current literature indicating that eyelid twitching is a benign condition that resolves spontaneously and is usually not associated with neurologic disease, there is no study investigating the relationship between digital screen time, uncorrected refractive error, IOP, and blood electrolyte levels with eyelid twitching [3]. This study aims to investigate the association between EM and digital screen time, uncorrected refractive error, IOP, and blood electrolyte levels in comparison with a healthy control group.

All patients were referred to the neurology clinic for evaluation for neurologic diseases. Following the neurologic evaluations, three patients with persistent headaches were referred for further investigation via brain magnetic resonance imaging. However, no pathological findings were identified. To minimize the influence of variables such as fatigue and coffee consumption, which have been linked to eyelid twitching in previous studies, participants with comparable daily sleep duration, working hours, and coffee consumption were included in the study [1,8]. 

In recent years, digital smart devices have assumed a significant role in our lives, with many individuals devoting a substantial portion of their leisure time to interacting with a small screen at close range. In particular, those who work in front of computers spend the majority of their waking hours in front of digital screens. The shift in work practices and remote work conditions that emerged during the pandemic period also led to an increase in digital screen time [9]. In this study, the average time spent in front of the screen during the day was found to be 6.88±2.01 hours in the EM group and 4.84±1.74 hours in the control group. The results indicated that the digital screen time was significantly higher in the EM group compared to the control group. In addition, a strong positive correlation was identified between digital screen time and the duration of eyelid twitching (p<0.001, r=0.670), indicating that the longer individuals spent in front of digital screens, the more likely they were to experience eyelid twitching. The quantity and intensity of light that our eyes are exposed to when we are in front of digital screens is typically considerably greater than it should be. As the amount of illuminance (i.e., the quantity of light) entering the eye increases, the orbicularis oculi muscle contracts, causing the eyelid to squint semi-autonomously. This action serves to reduce the amount of light entering the pupil. Furthermore, in situations requiring prolonged focus on a single point, such as driving or working in front of a digital screen, the number of blinks decreases [10,11]. Regular, rhythmic blinking is necessary for the orbicularis oculi muscle to relax sufficiently after contraction [12,13]. Electrophysiologic studies on the pathophysiology of myokymia have demonstrated the presence of undulatory muscle spasms and impaired muscular relaxation, despite the absence of any discernible abnormalities in the nervous system [14,15]. I think that the prolonged contraction of the orbicularis oculi muscle during screen time, in conjunction with the inability to relax the muscle sufficiently, may explain the observed relationship between digital screen time and the duration of eyelid twitching.

In the presence of uncorrected refractive error, the eyelids are squinted to create a pinhole effect and a clear image is attempted to be obtained [16]. According to the pathophysiologic mechanism of myokymia described above, I hypothesized that the presence of uncorrected refractive error and higher mean SE values may be present in patients with eyelid twitching. However, no significant difference was found between the mean SE values of the control group and the EM group in this study. The fact that the majority of both the patients in the EM group and the participants in the control group were emmetropes may be an explanation for this result. The results of future studies with a large number of participants with refractive error may serve to confirm my hypothesis, which is contrary to the results observed in this study.

A significant proportion of patients who consult an ophthalmologist with eyelid twitching express concern that this condition may be related to glaucoma. Upon questioning patients, it becomes evident that the source of their anxiety stems from misinformation obtained from social media or online research. Although eyelid twitching is not the primary symptom of glaucoma, some individuals with glaucoma may experience occasional eyelid spasms. To date, no study has been conducted in the literature investigating the relationship between eyelid twitching, glaucoma, and IOP. In this study, no significant difference was found between the mean IOP and C/D ratio of patients with eyelid twitching and healthy individuals in the control group.

Anecdotally, in patients presenting to ophthalmology outpatient clinics with prolonged eyelid twitching, there is an increasing tendency to report the use of various vitamins and supplements, with a notable prevalence of those containing Mg. Mg may play a role in reducing neuromuscular excitability. Mg preparations have been used in the treatment of cramps for years [17]. In recent years, Mg has become a prominent ingredient in nutritional supplements, with numerous salt derivatives of Mg being added to various products [18]. Many patients with eyelid twitching report using Mg preparations, often based on social media recommendations and advice from non-health professionals. In the present study, the blood Mg levels of participants in both the EM group and the control group were within the normal range. The mean blood Mg levels of both groups exhibited no significant difference.

The electrolytes Na and K play a crucial role in energy metabolism, affecting the function of our muscle cells, as well as all cells in the body. Together with the enzyme Na,K-ATPase, these two electrolytes facilitate the release of free energy required for the contraction and relaxation of muscle cells [19]. Irregularities in Na and K blood levels can result in conditions such as tetany and myoclonus in muscles [20]. In this study, no significant difference was observed between the mean blood Na and K levels of the patient and control groups. While the blood Na levels of 101 participants (98%) in the EM group were within the normal range, hypernatremia was identified in only two individuals (1.9%). All individuals exhibited blood K levels within the normal range.

Calcium ions (Ca^2+^) serve as the fundamental electrolyte that facilitates the functioning of all striated muscle cells in the human body. Ca^2+ ^ions bind to troponin on the actin filament in striated muscles, regulating the connection between myosin and actin and providing muscle movement [21]. Vitamin D deficiency is a relatively common condition in the community, and vitamin D deficiency causes hypocalcemia, which presents as neuromuscular excitability in the form of muscle twitching, spasms, tingling, and numbness [22]. In this study, the blood Ca levels were found to be low in only three patients (2.9%) with eyelid twitching, and hypocalcemia was found in two participants (1.9%) in the control group. No significant difference was observed between the blood Ca levels of the EM group and the control group.

The limitations of the study include the relatively small sample size, the fact that the study was conducted in a single center, and the data on the duration of digital screen time were based on the subjective statements of the participants. When determining digital screen time, participants were asked for a 14-day period, including two weekends, during which screen time on weekdays and off weekends could be different. Nevertheless, there may be periodic variations in screen time and future similar studies should include a longer period of assessment.

## Conclusions

In conclusion, the results of this study indicate that subjects with eyelid twitching exhibit significantly higher digital screen time. Consequently, prolonged digital screen time may be a contributing factor in the development of EM. In contrast, no association was observed between eyelid twitching and uncorrected refractive error, glaucoma, or blood electrolyte levels. Individuals presenting with prolonged eyelid twitching should be advised to limit their digital screen time.

## Appendices

**Table 3:** A translated form of the chart given to participants to record their daily digital screen time, sleep time, working time, and coffee consumption.

Days	Time spent with cell phones, tablets, and computers per day (hours)	Daily time spent asleep (hours)	Daily working hours at your job	Daily cofee consumption (cups/day)
1				
2				
3				
4				
5				
6				
7				
8				
9				
10				
11				
12				
13				
14				
